# Maternal Nutrition and Glycaemic Index during Pregnancy Impacts on Offspring Adiposity at 6 Months of Age—Analysis from the ROLO Randomised Controlled Trial

**DOI:** 10.3390/nu8010007

**Published:** 2016-01-04

**Authors:** Mary K. Horan, Ciara A. McGowan, Eileen R. Gibney, Jacinta Byrne, Jean M. Donnelly, Fionnuala M. McAuliffe

**Affiliations:** 1 UCD Obstetrics and Gynaecology, School of Medicine and Medical Science, University College Dublin, National Maternity Hospital, Dublin 2, Ireland; maryhoran21@hotmail.com (M.K.H.); cmcgowa@gmail.com (C.A.M.); jabyrne@nmh.ie (J.B.); donnelly.jean@gmail.com (J.M.D.); 2 School of Agriculture & Food Science, University College Dublin, Dublin 4, Ireland; Eileen.gibney@ucd.ie

**Keywords:** glycaemic index, maternal nutrition, offspring adiposity, childhood obesity, fetal programming

## Abstract

Childhood obesity is associated with increased risk of adult obesity and metabolic disease. Diet and lifestyle in pregnancy influence fetal programming; however the influence of specific dietary components, including low glycaemic index (GI), remains complex. We examined the effect of a maternal low GI dietary intervention on offspring adiposity at 6 months and explored the association between diet and lifestyle factors in pregnancy and infant body composition at 6 months. 280 6-month old infant and mother pairs from the control (*n* = 142) and intervention group (*n* = 138), who received low GI dietary advice in pregnancy, in the ROLO study were analysed. Questionnaires (food diaries and lifestyle) were completed during pregnancy, followed by maternal lifestyle and infant feeding questionnaires at 6 months *postpartum*. Maternal anthropometry was measured throughout pregnancy and at 6 months post-delivery, along with infant anthropometry. No difference was found in 6 months infant adiposity between control and intervention groups. Maternal trimester three GI, trimester two saturated fats and trimester one and three sodium intake were positively associated with offspring adiposity, while trimester two and three vitamin C intake was negatively associated. In conclusion associations were observed between maternal dietary intake and GI during pregnancy and offspring adiposity at 6 months of age.

## 1. Introduction

The *in utero* environment is important for fetal growth and development [1] as well as fetal programming [2,3]. Macrosomia has been found to result in greater risk of childhood obesity [4,5,6] and consequently adulthood overweight and obesity [7,8] as it is well established that overweight and obesity track from infancy throughout life [9,10,11]. The “Barker hypothesis” is the well-accepted association between small for gestational age infants and increased risk of overweight, obesity and metabolic syndrome in later life thought to occur due to fetal programming of a thrifty phenotype which is disadvantageous in an obesogenic environment [3,12]. Fetal programming of overweight and obesity is also thought to occur in large for gestational age infants with a J shaped curve of higher later Body Mass Index (BMI) at extremes of high and low birthweight observed [9,13,14,15]. Many maternal factors including pre-pregnancy BMI, gestational weight gain, and maternal lifestyle and diet during pregnancy have been found to be associated with offspring adiposity [16,17,18,19,20,21,22,23,24]. In addition to the *in utero* environment, later life adiposity is also known to be affected by the postnatal environment with formula fed infants growing faster than breastfed infants and at increased risk of overweight and obesity in childhood [25,26,27] while the results regarding persistence of a breastfeeding effect into adulthood are less definitive [28,29,30]. Greater catch-up growth in small for gestational age infants has also been found to increase later risk of excess adiposity [31,32,33,34] as have steeper growth trajectories (*i.e.*, velocity of infant weight gain) regardless of birthweight [35,36,37,38]. The postnatal environment is thought to influence adiposity by a myriad of mechanisms from nutrient and energy intake [39,40], satiety signalling [41], taste development [42], behaviour modelling by parents and siblings [43] to chemical compounds in breast milk [44] and bacterial colonisation of the gut [45]. Although there has been considerable research into the mechanisms involved in fetal programming of small for gestational age infants, there remains a paucity of data on the mechanisms and effects of fetal programming by the maternal environment in the case of overnutrition and diet quality with regard to large for gestational age infants.

There has been little research into the effect of low GI healthy eating interventions in pregnancy on maternal weight *postpartum* except in the case of gestational diabetes interventions which have shown little success after pregnancy [46,47,48]. Women from the ROLO (Randomised cOntrol trial of LOw glycaemic index diet *versus* no dietary intervention to prevent recurrence of fetal macrosomia) study were found to have reduced weight gain and greater reported dietary health behaviours such as food label reading diet at 3 months *postpartum* but there was no significant difference in weight or BMI between the control and intervention groups at this time point [49].

The aim of this study was to examine the effect of a low GI intervention in pregnancy on maternal and offspring anthropometry at 6 months *postpartum* as well as to explore associations between maternal characteristics and diet in pregnancy and infant size and adiposity at 6 months of age in a cohort of the ROLO study.

## 2. Methods

A total of 280 mother and infant pairs from the ROLO study [50] were included in this analysis. The ROLO study was a randomised control trial of 800 secundigravida women who had previously given birth to a macrosomic baby (>4 kg). The women were randomised to receive low glycaemic index (GI) dietary advice *versus* usual antenatal care, which did not involve dietary advice, to reduce recurrence of macrosomia. Detailed methodology and results of the ROLO study, which was carried out in the National Maternity Hospital, Ireland, have previously been published [50,51]. In brief; the primary outcome was birthweight which was not reduced and the secondary outcomes were gestational weight gain and glucose intolerance which were both reduced in the intervention group. Low GI dietary advice was given at week 14 of pregnancy and demographic, well-being and lifestyle questionnaires were returned by 28 weeks gestation. 3-day food diaries were completed during each trimester of pregnancy and used to determine the glycaemic index and glycaemic load of the women’s diets. The ROLO study found that the intervention group significantly lowered their glycaemic index (57.3 ± 4.0 *vs.* 56.0 ± 3.7, *p* < 0.001) following the intervention [50,51]. This study was conducted according to the guidelines laid down in the Declaration of Helsinki with institutional ethics approval from the National Maternity Hospital, Ireland. Informed written maternal consent was obtained from all participants both during pregnancy and at the 6 months follow-up appointment. Trial Registration with Current Controlled Trials ISRCTN54392969.

### 2.1. Inclusion and Exclusion Criteria

Participants were secundigravida women with a previous macrosomic baby (>4 kg). They were required to have sufficient literacy and English language fluency to understand the intervention and be capable of completing questionnaires. Women were only included if they had healthy, singleton pregnancies with no intrauterine growth abnormalities. All participants were invited to return for a follow-up appointment with their infant at 6–9 months *postpartum*.

### 2.2. Maternal Demographics, Lifestyle and Infant Feeding Practices

Of the 800 participants of the ROLO study, 280 (142 from the control and 138 from the intervention group) returned at 6 months of age and had completed food diaries throughout pregnancy as well as questionnaires in the first half of pregnancy exploring socioeconomic, socio-demographic, and lifestyle variables. Questions from SLAN (Survey of Lifestyle, Attitudes and Nutrition in Ireland) [52] relating to lifestyle habits were completed, including questions on physical activity, smoking, education and food label reading. Unfortunately, complete data was unavailable for some of the women for areas such as strenuous physical activity and breastfeeding duration due to incomplete questionnaires being returned. As this was a retrospective analysis, it was not possible to correct this issue.

### 2.3. Maternal and Infant Anthropometry

Maternal weight, height and mid-upper arm circumference were measured at the first antenatal consultation and BMI calculated. Maternal weight was also measured at each subsequent consultation and gestational weight gain calculated. Gestational weight gain data is not available for the full cohort as not all women returned for a final weight measurement very close to delivery. Maternal weight and mid-upper arm circumference were measured at 6 months *postpartum* and BMI was calculated.

Infant weight, length, mid-upper arm, abdominal, hip and thigh circumference, and biceps, triceps, subscapular and thigh skinfold measurements were taken at 6 months *postpartum*. Weight and length were measured for all 280 infants while other anthropometric measurements began to be taken later in the study and therefore complete data were available for 217 infants. World Health Orgaisation (WHO) growth standards were used to convert the infant measurements to z-scores which are adjusted for infant age and gender and give a measure of SDs away from the median [53,54]. Waist:hip, waist:length and triceps skinfold:subscapular skinfold ratios as well as sum of triceps and subscapular skinfold thicknesses and sum of all skinfold thicknesses were calculated in order to determine infant adiposity.

### 2.4. Maternal Dietary Intakes during Pregnancy

3-day food diaries were completed at each trimester of pregnancy and used to determine macro- and micro-nutrient intake as well as the glycaemic index and glycaemic load of the women’s diet. Macronutrients were expressed as a percentage of total energy while micronutrients were not adjusted for energy intake in order to determine the association of absolute micronutrient intake with offspring adiposity. Macro- and micro-nutrient intake in each trimester of pregnancy was examined separately. Underreporting was examined using Goldberg ratios, *i.e.*, the ratio of energy intake to estimated basal metabolic rate [55]. Basal metabolic rate was calculated using Schofield equations and a Goldberg ratio of ≤ 0.9 was used to identify definite under-reporters [55,56,57]. Analysis was carried out both including and excluding definite under-reporters.

Full methodology for entry and analysis of the dietary intake of participants has previously been published [51] but in brief, all food diaries were entered by a research dietitian with the use of the household measures and UK Food Standards Agency average portion sizes [58]. Food Diaries were analysed using Tinuviel WISP software (version 3.0, Tinuviel Software, Warrington, UK) in which the food composition tables used are derived from the 6th edition of McCance and Widdowson’s Food Composition Tables [59]. GI values were updated in WISP from 2002 values using the 2008 International Tables of Glycaemic Index Values and other more recently published GI values [60,61].

Information on maternal diet, *i.e.*, food diaries or food frequency questionnaires was not collected at 6 months *postpartum*. Information on infant feeding practices was collected at this time-point, *i.e.*, whether or not infant had ever been breastfed, the duration of breastfeeding, the age at which solids and other drinks were introduced.

### 2.5. Statistical Analysis

Statistical analysis was completed using SPSS (Statistical Package for the Social Sciences) software (version 20.0., IBM Corp, Armonk, NY, USA) Statistical analyses involved correlations, independent sample *t*-tests, ANOVA, ANCOVA, simple and multiple linear regression modelling and multiple logistic regression. Since there was no difference in infant anthropometry between the control and intervention groups, groups were combined for analysis but group was controlled for in all final models. Variables examined included macro- and micro-nutrient intake for each trimester, maternal height, weight and BMI during pregnancy and maternal weight and BMI at 6 months *postpartum*, gestational weight gain, change in weight from late pregnancy to 6 months *postpartum*, reported maternal physical activity, smoking and alcohol intake during pregnancy and at 6 months *postpartum*, maternal ethnicity, maternal age at delivery, marital/partner status, fasting glucose and oral glucose challenge test results during pregnancy and glycaemic index and load status during pregnancy. All variables were first examined using correlations. Variables that were found to be significantly associated with infant anthropometry at 6 months of age using correlations were further analysed using simple linear regression then input into final multiple regression models using a forced enter and backwards stepwise approach. This approach examined the association between the dependent and independent variables when all other variables in the model are controlled for, resulting in any non-significant variables being discarded from the model in a stepwise manner. Variables known to affect infant size (birthweight, education level as a marker of socioeconomic status, age in weeks at 6 months follow-up exam, infant gender and breastfeeding duration) were also controlled for using a forced enter multiple regression block in these models. Note: models predictive of infant anthropometric measurements for which z-scores were available were not adjusted for infant age and gender as these factors are taken into account when converting to z-scores. As mentioned, membership of the control or intervention group was controlled for in these models and definite maternal dietary underreporting, as determined using a Goldberg ratio ≤ 0.9, was also controlled for in these multiple linear regression models. Analysis was also carried out excluding definite under-reporters in order to ensure results were not altered by dietary underreporting. Multiple linear regression resulted in a best and final model and those that were statistically significant overall (*p* < 0.05) were chosen as those which best predicted neonatal anthropometric measurements. Maternal weight and BMI were compared between the control and intervention groups at 6 months *postpartum* using independent sample *t*-tests as was 6 months old infant anthropometry. Finally, logistic regression was used to determine factors associated with increased or decreased risk of infant being “at risk of overweight/overweight/obese” based on the WHO classification for BMI-for-age z-scores [54].

## 3. Results

### 3.1. Demographics, Lifestyle and Infant Feeding Practices

There was no difference in maternal background characteristics between the control and intervention groups during pregnancy or at 6 months *postpartum* in this cohort of women who returned for follow-up as illustrated in Table 1.

**Table 1:** Maternal characteristics during pregnancy and at 6 months and comparison of control and intervention (low glycaemic index diet) groups.

Background Maternal Characteristics	*n*	Intervention	Control	Total	*p*-Value
Age at Delivery (years)	256	33.18 ± 3.83	33.04 ± 4.14	33.11 ± 3.98	0.775
Height (cm)	280	165.12 ± 15.43	165.70 ± 6.76	165.41 ± 11.83	0.682
Weight booking (kg)	279	71.91 ± 12.93	71.81 ± 13.05	71.86 ± 12.97	0.947
BMI booking (kg/m^2^)	279	25.97 ± 4.27	26.16 ± 4.54	26.07 ± 4.40	0.717
Gestational weight gain (kg)	139	13.26 ± 4.48	13.77 ± 4.93	13.51 ± 4.70	0.525
Weight 6 months *postpartum* (kg)	213	74.96 ± 14.47	74.86 ± 12.89	72.45 ± 13.86	0.100
BMI 6 months *postpartum* (kg/m^2^)	213	27.26 ± 5.42	25.57 ± 4.63	26.38 ± 5.08	0.015
Duration of breastfeeding (wks)	166	4.25 ± 3.71	3.30 ± 4.15	3.78 ± 3.95	0.124
Days per week walking ≥30 min	224	3.54 ± 1.87	3.55 ± 1.75	3.54 ± 1.80	0.984
Strenuous PA early pregnancy (No. of 20 min intervals per week)	26	2.31 ± 1.20	1.60 ± 0.70	2.04 ± 1.08	0.101
Moderate PA early pregnancy (No. of 20 min intervals per week)	148	3.72 ± 2.76	2.96 ± 2.16	3.36 ± 2.51	0.066
Mild PA early pregnancy (No. of 20 min intervals per week)	211	4.19 ± 2.55	4.25 ± 2.50	4.22 ± 2.52	0.853
No. of min sitting/weekday baseline	236	393.75 ± 174.74	379.26 ± 179.68	386.32 ± 177.06	0.531
No. of min sitting/weekend day baseline	233	300.49 ± 124.19	304.67 ± 144.19	302.64 ± 134.59	0.813
No. of hours watching television/day baseline	249	1.76 ± 0.58	1.79 ± 0 .60	1.78 ± 0.59	0.753
Glucose booking	270	4.52 ± 0.36	4.53 ± 0.38	4.52 ± 0.37	0.838

Independent *t*-tests were used for this analysis. *p* < 0.05 was considered statistically significant (2-tailed significance). Abbreviations: PA: physical activity; mths: months; wks: weeks; No.: number.

### 3.2. Loss to Follow-up

Participants of the original ROLO study who returned for a 6 months follow-up appointment were significantly older (33.1 ± 4.0 *vs.* 32.4 ± 4.3 years, *p* = 0.029) and took part in more 20 min intervals of mild physical activity (4.2 ± 2.5 *vs.* 3.8 ± 2.3 intervals per week, *p* = 0.049). They had significantly higher infant birthweights (4.1 ± 0.5 *vs.* 4.0 ± 0.5 kg, *p* = 0.028) and length of gestation (282.2 ± 7.2 *vs.* 281.1 ± 9.0 days, *p* = 0.008) but not birthweight centile (centile 72.9 ± 24.4 *vs.* 71.8 ± 25.9, *p* = 0.574). The mothers had lower maternal weight, mid-upper arm circumference and BMI at booking (BMI 26.1 ± 4.4 *vs.* 27.1 ± 5.2 kg/m^2^, *p* = 0.008) and had a higher level of educational attainment (62% *vs.* 50% had achieved 3rd level education, *p* = 0.003). Their blood results showed an increased fasting glucose level at booking (4.5 ± 0.4 *vs.* 4.4 ± 0.4 mmol/L, *p* < 0.001) and a higher fasting glucose level at 28 weeks gestation (4.54 ± 0.69 *vs.* 4.45 ± 0.47 mmol/L, *p* = 0.049), as well as a higher glucose level following 28 week glucose challenge test (6.6 ± 1.7 *vs.* 6.3 ± 1.9 mmol/L, *p* = 0.032). A higher number of women were living with a partner (88% *vs.* 82% live with a partner, *p* = 0.029), and had a higher reported compliance with low GI intervention (81% *vs.* 70% reported following low GI diet most of the time, *p* = 0.033) than those who did not attended follow-up assessments. Their reported dietary intakes were similar except that those who returned for the 6 months assessment had higher energy intakes in trimesters 1 and 3, and higher energy corrected micronutrient intakes as illustrated in supplemental Table S1. Their level of definite underreporting (*i.e.*, Goldberg ratio ≤ 0.9) was not significantly different from those who did not attend the 6 months follow-up assessment (*p* = 0.603).

### 3.3. Maternal and Infant Anthropometry

At baseline there was no difference between the control and intervention groups for maternal BMI (26.0 ± 4.3 *vs.* 26.2 ± 4.5 kg/m^2^, *p* = 0.717) or mid-upper arm circumference (28.9 ± 2.9 *vs.* 29.4 ± 3.3, *p* = 0.186). As mentioned the intervention group had a lower level of gestational weight gain than the control group in the overall ROLO cohort however in the subgroup who took part at 6 months *postpartum* there was no difference in gestational weight gain (13.3 ± 4.5 *vs.* 13.8 ± 4.9 kg, *p* = 0.525). Maternal weight at 6 months was not significantly different between the intervention and control groups (74.9 ± 14.5 *vs.* 74.9 ± 12.9 kg respectively, *p* = 0.10); however, maternal BMI at 6 months *postpartum* was found to be significantly greater in the control group (27.3 ± 5.4 *vs.* 25.6 ± 4.6, *p* = 0.015). There were 3 women in the intervention group with BMI > 40 kg/m^2^ compared with only 1 in the control group. There was no difference in infant anthropometry between the control and intervention groups at birth in the original ROLO cohort except thigh circumference which was smaller in the intervention group as mentioned [62]. At 6 months, there was no difference in any infant anthropometric measure between the control and intervention groups (Table 2). BMI-for age classification revealed that at 6 months; 224 infants (83.3%) were not overweight or at risk of overweight, 34 (12.6%) were at risk of overweight, 9 (3.3%) were overweight and 2 (0.7%) were obese. Again, there was no difference in BMI-for age classification between the control and intervention groups (*p* = 0.636). Data on maternal and infant anthropometry are displayed in Table 1 and Table 2 respectively.

**Table 2:** 6 months infant anthropometry and comparison of control and intervention (low glycaemic index diet) groups.

Infant Anthropometry	*n*	Intervention	Control	Total	*p*-Value
Age at 6 mths follow-up appointment (weeks)	280	28.16 ± 9.95	30.80 ± 15.53	29.50 ± 13.14	0.098
Weight-for-Length z-score at 6 mths	280	0.34 ± 1.90	0.1241 ± 1.05	0.23 ± 1.52	0.241
Weight at 6 mths (kg)	280	8.61 ± 1.77	8.34 ± 1.09	8.48 ± 1.47	0.127
Weight-for-age z-score at 6 mths	280	0.93 ± 1.59	0.65 ± 1.00	0.79 ± 1.33	0.075
Length at 6 mths (cm)	280	70.02 ± 3.36	69.62 ± 2.90	69.82 ± 3.13	0.286
Length-for-age z-score at 6 mths	280	1.39 ± 1.39	1.17 ± 1.09	1.27 ± 1.24	0.150
BMI-for-age z-score at 6 mths	280	0.20 ± 1.95	−0.0109 ± 1.05	0.09 ± 1.56	0.263
MUAC-for-age z-score at 6 mths (cm)	280	15.31 ± 1.72	15.44 ± 1.30	15.38 ± 1.52	0.467
MUAC-for-age z-score at 6 mths	280	1.00 ± 1.60	1.15 ± 1.09	1.08 ± 1.36	0.393
Triceps skinfold-for-age z-score at 6 mths	217	0.08 ± 1.11	−0.0175 ± 1.16	0.03 ± 1.13	0.531
Subscapular skinfold-for-age z-score at 6 mths	218	0.73 ± 1.05	0.41 ± 1.24	0.56 ± 1.16	0.051
Abdominal Circumference at 6 mths (cm)	280	43.94 ± 4.03	44.09 ± 3.51	44.02 ± 3.77	0.747
Chest Circumference at 6 mths (cm)	279	44.89 ± 3.08	44.54 ± 2.65	44.72 ± 2.87	0.310
Hip Circumference at 6 mths (cm)	279	44.64 ± 4.11	44.46 ± 3.45	44.55 ± 3.78	0.697
Thigh Circumference at 6 mths (cm)	280	24.09 ± 3.24	23.96 ± 2.64	24.03 ± 2.95	0.714
Biceps skinfold at 6 mths (mm)	217	9.01 ± 2.02	9.02 ± 2.01	9.01 ± 2.01	0.960
Thigh skinfold at 6 mths (mm)	217	11.95 ± 2.51	11.70 ± 3.12	11.82 ± 2.84	0.521
Sum of all skinfolds at 6 mths (mm)	217	38.61 ± 5.47	37.76 ± 6.24	38.17 ± 5.88	0.286
Sum Triceps and Subscapular skinfolds at 6 mths (mm)	217	17.66 ± 2.70	17.04 ± 3.10	17.34 ± 2.92	0.119
Triceps:Subscapular skinfold ratio at 6 mths	217	0.91 ± 0.23	0.87 ± 0.24	0.89 ± 0.23	0.195
Waist:Hip Circumference ratio at 6 mths	279	0.99 ± 0.80	0.99 ± 0.08	0.99 ± 0.08	0.457
Waist Circumference:Length ratio at 6 mths	280	0.63 ± .051	0.63 ± .048	0.63 ± 0.05	0.328

Independent *t*-tests were used for this analysis. *p* < 0.05 was considered statistically significant (2-tailed significance). Abbreviations: MUAC: mid-upper arm circumference; mths: months.

### 3.4. Maternal Dietary Intakes during Pregnancy

Maternal dietary intakes during pregnancy are displayed in supplemental Table S2 with a comparison of control and intervention groups. Following low GI intervention similar to the overall ROLO cohort, the intervention group had lower energy intake, higher protein intake, lower total carbohydrate intake and GI and GL in trimester 2 and 3 [50,51]. Rates of definite underreporting of dietary intake in this cohort was 12.9% similar to levels of the overall ROLO cohort (*i.e.*, 12.4%) and there was no difference between the control and intervention groups (9.1% *vs.* 17.2% respectively, *p* = 0.060). Finally, although detailed dietary data were not available for this cohort at 6 months *postpartum*, there was no difference in reported numbers of mothers reading food labels (*p* = 0.682) or number of occasions of consumption of fried food per week (*p* = 0.299) between the control and intervention groups.

### 3.5. Association of Maternal Characteristics with Infant Anthropometry

Factors associated with infant anthropometry, including maternal anthropometry and lifestyle, are displayed in final multiple regression models in Table 3, while unadjusted models are displayed in Table S3. Maternal anthropometry at 6 months *postpartum* was not associated with offspring anthropometry. In terms of lifestyle; gestational weight gain was positively associated with 6 months old mid-upper arm circumference (B = 0.059, *p* = 0.002) while the number of 20 min intervals of moderate maternal physical activity was positively associated with 6 months old subscapular skinfold thickness for age z-score (B = 0.115, *p* = 0.013). No association between change in maternal weight from late pregnancy to 6 months *postpartum* and any measure of offspring anthropometry was observed.

**Table 3:** Maternal and paternal characteristics and maternal nutrient intakes associated with 6 months old offspring adiposity-adjusted analysis *.

Model	*Variable*			*Total Model*	
	B	SEB	*p*-Value	*R*^2^_adj_	*p*-Value
**Weight for length z-score**				0.079	0.001
Trimester 1 sodium	0.0003	0.0001	0.003		
**Weight for age z-score**				0.141	<0.001
Trimester 2 saturated fatty (%TE)	0.075	0.027	0.005		
**Length for age z-score**				0.186	<0.001
Trimester 1 carotene	9.63E−05	0.00004	0.013		
Trimester 1 PUFA (%TE)	0.159	0.049	0.002		
**BMI for age z-score**				0.071	0.002
Trimester 1 sodium	0.0003	0.0001	0.003		
**MUAC-for-age z-score**				0.182	0.001
Trimester 3 sodium	0.0003	0.0001	0.009		
Gestational weight gain (kg)	0.059	0.018	0.002		
**Triceps skinfold-for-age z-score**				0.088	0.003
Trimester 3 vitamin C	−0.005	0.003	0.035		
Trimester 3 glycaemic index	0.053	0.023	0.023		
**Subscapular skinfold-for-age z-score**				0.071	0.045
Moderate PA baseline (number of 20 min intervals/day)	0.115	0.045	0.013		
**Thigh circumference**				0.099	0.001
Trimester 1 sodium	0.001	0.0003	0.013		
Trimester 1 carotene	0.0002	0.00008	0.038		
Trimester 3 carbohydrate (%TE)	−0.081	0.034	0.019		
**Biceps skinfold**				0.109	0.003
Trimester 1 thiamine	0.603	0.207	0.004		
Baseline minutes sitting/weekday	0.002	0.001	0.064		
Trimester 3 glycaemic index	0.121	0.04	0.003		
**Sum of triceps and subscapular skinfolds**				0.067	0.015
Trimester 2 vitamin C	−0.015	0.005	0.007		
**Waist:Hip circumference ratio**				0.163	<0.001
Trimester 1 vitamin B6	−0.028	0.012	0.019		
Trimester 3 potassium	3.01E−05	8.39E−06	<0.001		
Trimester 1 glycaemic index	−0.004	0.001	0.004		
Trimester 2 saturated fat (%TE)	0.006	0.002	<0.001		

Backwards stepwise multiple linear regression was used for this analysis * adjusted for well-established influences on neonatal size. Group affiliation was also included in all models (*i.e.*, control or intervention). Z-score values were adjusted for birthweight, education, intervention group, breastfeeding and definite underreporting (Goldberg ratio ≤ 0.9). Non-z-score values were adjusted for birthweight, education, infant age at measurement, infant gender, intervention group, breastfeeding and definite underreporting (Goldberg ratio ≤ 0.9). *p* < 0.05 was considered statistically significant. Abbreviations: MUAC: mid-upper arm circumference; PA: physical activity; %TE: percentage of total energy; BMI: body mass index; PUFA: poly-unsaturated fatty acid; SEB: the standard error of the computed value of B.

### 3.6. Association of Maternal Dietary Intake during Pregnancy and Infant Anthropometry

Factors associated with infant anthropometry, including maternal macro- and micro-nutrient intake, are displayed in final multiple linear regression models in Table 3. Several dietary factors were associated recurrently with offspring adiposity. Maternal trimester 1 sodium intake was positively associated with 6 months old weight for length z-score (B = 0.0003, *p* = 0.003), BMI-for-age z-score (B = 0.0003, *p* = 0.003) and thigh circumference (0.001, 0.013) while trimester 3 sodium intake was positively associated with mid-upper arm circumference-for-age z-score (B = 0.0003, *p* = 0.009). Maternal trimester 2 saturated fat intake was positively associated with 6 months old weight-for-age z-score (B = 0.075, *p* = 0.005) and waist: hip circumference ratio (B = 0.006, *p* < 0.001). Maternal trimester 3 glycaemic index was positively associated with 6 months old triceps skinfold thickness for age z-score (B = 0.053, *p* = 0.023) and biceps skinfold thickness (B = 0.121, *p* = 0.003) while trimester 1 GI was negatively associated with waist:hip circumference ratio (B = −0.004, *p* = 0.004). Maternal trimester 3 vitamin C intake was negatively associated with 6 months old triceps-for-age skinfold thickness z-score (B = −0.005, *p* = 0.035) and, finally, trimester 2 vitamin C was negatively associated with the sum of the 6 months old triceps and subscapular skinfolds (B = −0.015, *p* = 0.007).

When BMI for age z-score was categorised into the dichotomies “not overweight or at risk of overweight” and “at risk of overweight or overweight or obese” simple logistic regression revealed a significant positive association between maternal trimester 1 sodium intake, trimester 1 vitamin B12 and trimester 1 retinol intake and a negative association with trimester 2 potassium and trimester 2 carotene intake. When these factors were further analysed by fitting a multivariable logistic regression model which significantly predicted risk of being “at risk of overweight/overweight/obese” (final model *p* < 0.001), a significant association remained only for trimester 1 sodium, trimester 2 carotene and trimester 1 vitamin B12 intake. Every 1 g greater maternal intake of sodium in trimester 1 was associated with a 1.7 fold increased risk of being at risk of the infant being overweight/overweight/obese (OR = 1.712, 95% CI: 1.13–2.633, *p* = 0.014) while every 1 mg greater maternal intake of carotene in trimester 2 was associated with a reduction in the risk of being at risk of overweight/overweight/obese (OR = 0.849, 95% CI: 0.739–0.976, *p* = 0.022). Finally, every 1 μg greater intake of vitamin B12 in trimester 1 was associated with a 2.5 fold increased risk of being at risk of overweight/overweight/obese (OR = 2.539, 95% CI: 1.301–4.958, *p* = 0.006).

## 4. Discussion

The main findings of this paper were that there was no significant difference in 6 months old anthropometry between the control and intervention group. Maternal GI as well as intake of several specific macro- and micro-nutrients during pregnancy was associated with 6 months old adiposity *i.e.*, maternal trimester 3 glycaemic index was positively associated with 6 months old triceps skinfold thickness for age z-score and biceps skinfold thickness while trimester 1 GI was negatively associated with waist:hip circumference; maternal trimester 2 saturated fat intake was positively associated with 6 months old weight-for-age z-score and waist:hip circumference ratio; maternal trimester 1 sodium intake was positively associated with 6 months old weight for length z-score, BMI-for-age z-score and thigh circumference while trimester 3 sodium intake was positively associated with mid-upper arm circumference-for-age z-score while every 1 g greater maternal intake of sodium in trimester 1 was associated with a 1.7 fold increased risk of the infant being at risk of overweight/overweight/obese. Finally, maternal trimester 3 vitamin C intake was negatively associated with 6 months old triceps-for-age skinfold thickness z-score and, finally, trimester 2 vitamin C was negatively associated with the sum of the 6 months old triceps and subscapular skinfolds. On the maternal side maternal weight and BMI were higher in the intervention than the control group.

The finding that maternal BMI was significantly higher in the intervention group at 6 months of age was unexpected. Very few studies have examined the effect of low GI diet during pregnancy on maternal diet or weight *postpartum* except two studies of euglycaemic women [49,63] and three studies of women with gestational diabetes [46,47,48]. While the study by Moses *et al.* [64] did not examine maternal anthropometry at 2 years *postpartum*, it found that women had reverted to pre-intervention dietary intakes. A previous follow-up of the ROLO study cohort at 3 months *postpartum* found no significant difference in weight or BMI between the control and intervention groups although the intervention group had significantly lower gestational weight gain and had gained significantly less weight overall from early pregnancy to 3 months *postpartum* [49]. However, dietary intake was not examined at 3 months *postpartum*. Studies of women with gestational diabetes have observed poor adherence to dietary change *postpartum* with one study finding that low GI dietary advice *vs.* traditional high fibre advice resulted in no significant difference in maternal weight at 3 months *postpartum* [48], another finding that healthy dietary changes were not sustained at 6 months *postpartum* [46] and another finding that although women reported being concerned about developing type II diabetes, more women had gained weight than had lost weight at 11–42 months *postpartum* although the number of women reporting consuming a high fat diet had decreased from that reported pre-pregnancy [47]. The finding of the current study that women in the intervention group had higher BMI at 6 months *postpartum* may be a chance finding as, unlike the total cohort, the intervention group of this follow-up cohort did not have significantly lower gestational weight gain than the control group and there was a high percentage of women lost to follow-up as unfortunately the 6 months study was not possible to begin until many of the infants were over 9 months of age and no longer eligible. On further examination, it was also noted that there were 3 women in the intervention group with BMI > 40 kg/m^2^ and only 1 in the control group which may have skewed the data. Unfortunately, detailed dietary data was not collected at 6 months *postpartum* so it was not possible to determine whether the increased BMI in the intervention group was due to increased dietary intake or whether women continued to adhere to a low GI diet or had reverted to pre-pregnancy dietary habits. Further research into the effect of low GI in pregnancy on *postpartum* BMI will be necessary to elucidate this.

While there was no difference between the control and intervention groups in 6 months old anthropometry due to the low GI dietary intervention in pregnancy, GI was associated with several 6 months old anthropometric measurements. Maternal trimester 3 glycaemic index was positively associated with 6 months old triceps skinfold thickness for age z-score and biceps skinfold thickness while trimester 1 GI was negatively associated with waist:hip circumference ratio, a measure of central adiposity. While there has been some interest in the effect of maternal dietary GI on offspring macrosomia [50,63,65,66,67], there is little data available on the effect on offspring adiposity in infancy or childhood and the data remains unclear. The only other randomised control trial of low GI in pregnancy that carried out a follow-up *postpartum* was the study by Moses *et al.* [63] which found that low GI dietary intervention in pregnancy reduced risk of large for gestational age births, unlike our findings from the ROLO study [50]. However, there was no difference between the control and intervention group in infant size at 2 years of age similar to our findings at 6 months of age [65]. This follow-up by Moses *et al.* [64] consisted of only 43 mother and child pairs which is likely to have affected the statistical power of the results however it did find that large for gestational age infants (originally *n* = 10 in the control group *vs. n* = 1 in the intervention group) had significantly higher weight but not height at 2 years of age. We previously found that there was a trend towards a positive association between trimester 2 GI and central adiposity at birth in this cohort [20]. A recently published study by Okubo [22] has found that maternal GI in early pregnancy was positively associated with offspring fat mass at 4 and at 6 years of age but not at birth. Okubo *et al.* also observed an association between maternal GI in late pregnancy and offspring fat mass at 4 and 6 years of age however this effect did not persist once confounders were controlled for [22]. Observational studies not focusing specifically on GI have also found associations with offspring adiposity; a study by Murrin *et al.* [68] found that maternal sugar intake in early pregnancy was significantly positively associated with offspring adiposity at 5 years of age, a recent study by Pereira-de-Silva *et al.* [69] found that maternal carbohydrate intake during pregnancy was positively associated with neonatal adiposity similarly to a study by Moore *et al.* [70] which found that maternal carbohydrate intake in early and late pregnancy was positively associated with neonatal ponderal index.

Maternal trimester 2 saturated fat intake was positively associated with 6 months old weight-for-age z-score and waist:hip circumference ratio, a measure of central adiposity. These findings are similar to those of a recent study by Murrin *et al.* [68] which found that maternal saturated fat intake in the first trimester of pregnancy was positively associated with offspring overweight and obesity at 5 years of age. Ladino *et al.* [71] also found that maternal fat intake was positively associated with offspring adiposity up to 18 months. We also previously observed a similar positive association at birth between neonate central adiposity and maternal trimester 3 SFA intake [20]. However, not all studies have observed an association between maternal macronutrient intake and offspring adiposity [72,73].

This study found that maternal sodium intake was positively associated with several measures of offspring adiposity at 6 months of age *i.e.*, trimester 1 sodium intake was positively associated with 6 months old weight-for-length z-score, BMI-for-age z-score and thigh circumference while trimester 3 sodium intake was positively associated with mid-upper arm circumference-for-age z-score. In addition, maternal sodium intake was associated with increased risk of being in an above normal World Health Organisation BMI-for-age category at 6 months. Although there is very little data available in the literature on the association between sodium intake during pregnancy and offspring adiposity, our findings are in agreement with those of Ladino *et al.* [71] who found that infant adiposity up to 18 months of age was positively associated with maternal sodium intake as well as sugar and fat intake during trimester 3. Our findings in offspring at 6 months of age are also similar to our findings at birth that neonatal central adiposity was positively associated with maternal sodium intake in trimester 1 of pregnancy. It is also likely that maternal sodium intake is acting as a marker of a maternal diet high in processed foods, cereals and baked goods which have been found to provide the greatest contribution to sodium intake in European and North American populations [74] while in Ireland the greatest contributors have been found to be breads and cured and processed meats [75]. Processed foods and salty snacks are highly palatable, contain high levels of energy and fat and may reduce endogenous satiety signalling, and therefore have been linked to global overweight and obesity [76]. However, discretionary addition of salt in cooking or at the table was not recorded in this study and therefore could not be accounted for.

Finally, maternal trimester 2 vitamin C intake was negatively associated with the sum of the 6 months old triceps and subscapular skinfolds, a measure of overall adiposity, while maternal trimester 3 vitamin C intake was negatively associated with 6 months old triceps-for-age skinfold thickness z-score. While micronutrients may act simply as markers of overall diet, lifestyle and health consciousness obscuring the relationship between their mechanisms of action and health outcomes [77], the multiple regression models used in this analysis were controlled for maternal macronutrient intake in order to reduce this effect. Of course, these results are observational and therefore it is impossible to assign causality.

### Limitations

One limitation of this study was that it was not possible to examine discretionary sodium addition in cooking and at the table in this analysis and the limitations of any salt analysis in the absence of urinary sodium studies should also be noted as it has been found that while the 3-day food diary gives the best subjective estimate of usual sodium intake, it underestimates in comparison to 24 h urine analysis [78]. The limitations of using food diaries in general should also be noted as participants may inaccurately report or inadvertently change their dietary habits while keeping a dietary record. A further limitation is that data from this cohort may not be generalizable to other populations as this cohort had a high level of macrosomia and concomitant risk of offspring obesity. In addition, there is likely some responder bias as those that returned for follow-up appear to be a more health-conscious group which may reflect the low levels of offspring overweight and obesity. This study had a high rate of loss to follow-up due to a delay in commencing 6 months follow-up data collection as the study was not originally designed to be continued beyond 3 months *postpartum* resulting in many of the infants no longer being within the eligible age-group. However, the loss to follow-up was therefore largely logistic rather than to do with participant reluctance to return, reducing the level of resulting bias and this study still involved a large cohort particularly suitable for examination of the effect of maternal diet and lifestyle during pregnancy on offspring adiposity. This study describes new and novel data from the ROLO study on the relationship between maternal diet and 6 months old offspring growth and adiposity, which is extremely valuable in light of the significant health issue which childhood obesity poses, the paucity of data in this area, and the need for interventions, including antenatal interventions to combat this issue. It is crucial to describe data supporting relationships between maternal diet, and lifestyle, particularly with an emphasis on modifiable factors, in order to allow appropriate interventions to be developed.

## 5. Conclusions

There was no effect of a low GI intervention in pregnancy on offspring adiposity. Maternal dietary intake throughout pregnancy, in particular saturated fat and sodium intake and late pregnancy GI were positively associated with infant adiposity at 6 months of age. While further research is necessary to determine optimal diet in pregnancy, improving maternal nutrition may be a simple public health intervention to reduce childhood obesity.

## Supplementary Materials

Online Supplemental Material Submitted: The following are available online at http://www.mdpi.com/2072-6643/8/1/7/s1, Table S1: Difference in dietary intake during pregnancy between women who returned for follow-up at 6 months postpartum and those who were lost to follow-up; Table S2: Maternal dietary macro- and micro-nutrient intakes, glycaemic index and glycaemic load during each trimester of pregnancy and comparison of control and intervention (low glycaemic index) groups, Table S3: Maternal characteristics and nutrient intakes associated with neonatal anthropometry unadjusted analysis.
