# Supplementary material for: Maternal Nutrition and Glycaemic Index during Pregnancy Impacts on Offspring Adiposity at 6 Months of Age—Analysis from the ROLO Randomised Controlled Trial
Source: Nutrients. 2016 Jan 4;8(1):7. doi: 10.3390/nu8010007 (PMC4728621; doi:10.3390/nu8010007)
Supplement: Supplementary file 1 [file nutrients-08-00007-s001.docx]

Supplementary Materials: Maternal Nutrition and Glycaemic Index during Pregnancy Impacts on Offspring Adiposity at 6 Months of Age—Analysis from the ROLO Randomised Controlled Trial

Mary K. Horan, Ciara A. McGowan, Eileen R. Gibney, Jacinta Byrne, Jean M. Donnelly and Fionnuala M. McAuliffe

**Table S1.** Difference in dietary intake during pregnancy between women who returned for follow-up at 6 months postpartum and those who were lost to follow-up.

| **Dietary Variable** | **Returned for Follow-up** | **Lost to Follow-up** | ***p*-Value** |
| --- | --- | --- | --- |
|  | **Mean ± SD** | **Mean ± SD** |  |
| Trimester 1 glycaemic Index | 58.0 ± 4.2 | 57.1 ± 4.0 | 0.021 |
| Trimester 1 energy (kcal/day) | 1892 ± 295 | 1817 ± 477 | 0.048 |
| Trimester 3 energy (kcal/day) | 1932 ± 436 | 1855 ± 466 | 0.047 |
| Trimester 1 iodine (ug/10 MJ) | 175.1 ± 69.5 | 159.9 ± 76.8 | 0.017 |
| Trimester 1 vitamin B12 (ug/10 MJ) | 2.2 ± 0.6 | 2.0 ± 0.6 | 0.007 |
| Trimester 1 folate (ug/10 MJ) | 366.2 ± 122.4 | 342.4 ± 110.9 | 0.017 |
| Trimester 2 potassium (mg/10 MJ) | 3635.6 ± 674.1 | 3487.5 ± 631.2 | 0.008 |
| Trimester 2 magnesium (mg/10 MJ) | 331.9 ± 62.4 | 321.4 ± 62.5 | 0.049 |
| Trimester 2 iron (mg/10 MJ) | 14.8 ± 4.1 | 14.1 ± 3.4 | 0.026 |
| Trimester 2 iodine (ug/10 MJ) | 181.8 ± 75.5 | 166.9 ± 74.2 | 0.020 |
| Trimester 2 vitamin K (ug/10 MJ) | 153.1 ± 98.7 | 136.9 ± 84.1 | 0.038 |
| Trimester 2 vitamin B12 (ug/10 MJ) | 2.2 ± 0.6 | 2.1 ± 0.6 | 0.030 |
| Trimester 2 biotin (ug/10 MJ) | 37.7 ± 11.1 | 35.6 ± 10.8 | 0.022 |
| Trimester 3 carotene (ug/10 MJ) | 4752.9 ± 3839.3 | 4082.0 ± 3473.9 | 0.032 |
| Trimester 3 biotin (ug/10 MJ) | 38.6 ± 12.7 | 36.3 ± 11.1 | 0.023 |

Independent sample t-tests were used for this analysis (statistically significant values only included). *p* < 0.05 considered statistically significant.

**Table S2.** Maternal dietary macro- and micro-nutrient intakes, glycaemic index and glycaemic load during each trimester of pregnancy and comparison of control and intervention (low glycaemic index) groups.

| **Nutrient** | ***n*** | **Intervention Mean ± SD** | **Control Mean ± SD** | **Total Mean ± SD** | ***p*-Value** |
| --- | --- | --- | --- | --- | --- |
| Trimester 1 Energy Intake (kcal/day) | 248 | 1848.28 ± 347.36 | 1931.08 ± 430.04 | 1892.03 ± 394.61 | 0.099 |
| Trimester 2 Energy Intake (kcal/day) | 253 | 1775.89 ± 396.60 | 1961.94 ± 376.43 | 1872.96 ± 396.53 | 0.000 |
| Trimester 3 Energy Intake (kcal/day) | 254 | 1858.04 ± 410.14 | 2000.11 ± 449.77 | 1931.87 ± 436.18 | 0.009 |
| Trimester 1 Protein Intake (%TE) | 248 | 17.48 ± 3.15 | 16.47 ± 2.93 | 16.95 ± 3.07 | 0.009 |
| Trimester 2 Protein Intake (%TE) | 253 | 18.32 ± 3.15 | 16.36 ± 2.72 | 17.30 ± 3.09 | 0.000 |
| Trimester 3 Protein Intake (%TE) | 254 | 17.91 ± 3.15 | 16.59 ± 3.13 | 17.22 ± 3.21 | 0.001 |
| Trimester 1 Carbohydrate Intake (%TE) | 248 | 50.57 ± 6.71 | 50.79 ± 6.15 | 50.68 ± 6.41 | 0.785 |
| Trimester 2 Carbohydrate Intake (%TE) | 253 | 48.79 ± 5.71 | 50.90 ± 5.59 | 49.89 ± 5.73 | 0.003 |
| Trimester 3 Carbohydrate Intake (%TE) | 254 | 48.57 ± 5.49 | 50.50 ± 6.21 | 49.57 ± 5.94 | 0.009 |
| Trimester 1Total Fat Intake (%TE) | 248 | 35.02 ± 5.61 | 35.78 ± 5.39 | 35.42 ± 5.49 | 0.276 |
| Trimester 2 Total Fat Intake (%TE) | 253 | 35.81 ± 5.13 | 35.82 ± 5.22 | 35.82 ± 5.17 | 0.993 |
| Trimester 3 Total Fat Intake (%TE) | 254 | 36.34 ± 5.34 | 35.90 ± 5.51 | 36.11 ± 5.42 | 0.519 |
| Trimester 1 Saturated Fat Intake (%TE) | 248 | 13.26 ± 2.81 | 13.82 ± 3.09 | 13.56 ± 2.97 | 0.138 |
| Trimester 2 Saturated Fat Intake (% TE) | 253 | 13.33 ± 3.15 | 13.75 ± 2.89 | 13.55 ± 3.02 | 0.262 |
| Trimester 3 Saturated Fat Intake (%TE) | 254 | 13.94 ± 2.93 | 13.86 ± 3.15 | 13.90 ± 3.04 | 0.845 |
| Trimester 1 Monounsaturated Fat Intake (%TE) | 248 | 11.06 ± 2.44 | 11.38 ± 2.36 | 11.23 ± 2.40 | 0.293 |
| Trimester 2 Monounsaturated Fat Intake (%TE) | 253 | 11.34 ± 2.33 | 11.34 ± 2.21 | 11.34 ± 2.26 | 0.990 |
| Trimester 3 Monounsaturated Fat Intake (%TE) | 254 | 11.47 ± 2.45 | 11.38 ± 2.25 | 11.42 ± 2.34 | 0.762 |
| Trimester 1 Polyunsaturated fat Intake (%TE) | 248 | 5.92 ± 1.93 | 5.56 ± 1.81 | 5.73 ± 1.87 | 0.133 |
| Trimester 2 Polyunsaturated Fat Intake (%TE) | 253 | 5.94 ± 1.58 | 5.66 ± 1.68 | 5.79 ± 1.64 | 0.169 |
| Trimester 3 Polyunsaturated fat Intake (%TE) | 254 | 5.76 ± 1.64 | 5.43 ± 1.76 | 5.59 ± 1.71 | 0.129 |
| Trimester 1 Glycaemic Index | 248 | 57.85 ± 4.41 | 58.07 ± 4.03 | 57.97 ± 4.21 | 0.689 |
| Trimester 2 Glycaemic Index | 253 | 56.19 ± 4.02 | 57.72 ± 3.84 | 56.99 ± 3.99 | 0.003 |
| Trimester 3 Glycaemic Index | 254 | 56.21 ± 3.87 | 57.75 ± 4.09 | 57.02 ± 4.05 | 0.003 |
| Trimester 1 Glycaemic Load | 248 | 135.24 ± 32.30 | 142.68 ± 39.91 | 139.14 ± 36.60 | 0.111 |
| Trimester 2 Glycaemic Load | 253 | 121.50 ± 29.53 | 143.79 ± 31.19 | 133.19 ± 32.34 | 0.000 |

**Table S2.** *Cont.*

| **Nutrient** | ***n*** | **Intervention Mean ± SD** | **Control Mean ± SD** | **Total Mean ± SD** | ***p*-Value** |
| --- | --- | --- | --- | --- | --- |
| Trimester 3 Glycaemic Load | 254 | 127.12 ± 27.76 | 146.43 ± 41.57 | 137.25 ± 36.90 | 0.000 |
| Trimester 1 Sodium (mg) | 248 | 2563.08 ± 708.12 | 2779.33 ± 769.50 | 2677.31 ± 747.56 | 0.023 |
| Trimester 2 Sodium (mg) | 253 | 2484.93 ± 717.55 | 2694.74 ± 850.02 | 2594.39 ± 794.87 | 0.036 |
| Trimester 3 Sodium (mg) | 254 | 2653.61 ± 707.39 | 2776.57 ± 772.10 | 2717.51 ± 742.82 | 0.188 |
| Trimester 1 Potassium (mg) | 248 | 2882.69 ± 688.32 | 2748.10 ± 698.27 | 2811.60 ± 695.45 | 0.128 |
| Trimester 2 Potassium (mg) | 253 | 2835.20 ± 682.06 | 2816.46 ± 672.20 | 2825.42 ± 675.65 | 0.826 |
| Trimester 3 Potassium (mg) | 254 | 2808.32 ± 672.01 | 2917.82 ± 705.17 | 2865.22 ± 690.26 | 0.207 |
| Trimester 1 Calcium (mg) | 248 | 935.63 ± 318.17 | 930.87 ± 268.20 | 933.1173 ± 292.24 | 0.898 |
| Trimester 2 Calcium (mg) | 253 | 924.01 ± 325.68 | 932.89 ± 270.61 | 928.65 ± 297.65 | 0.813 |
| Trimester 3 Calcium (mg) | 254 | 979.46 ± 370.22 | 954.03 ± 276.57 | 966.24 ± 324.53 | 0.534 |
| Trimester 1 Magnesium (mg) | 248 | 262.32 ± 60.90 | 248.21 ± 61.16 | 254.87 ± 61.32 | 0.070 |
| Trimester 2 Magnesium (mg) | 253 | 264.09 ± 67.28 | 253.17 ± 60.08 | 258.39 ± 63.73 | 0.174 |
| Trimester 3 Magnesium (mg) | 254 | 268.47 ± 69.77 | 258.99 ± 64.83 | 263.54 ± 67.28 | 0.263 |
| Trimester 1 Iron (mg) | 248 | 11.47 ± 2.98 | 11.80 ± 3.99 | 11.64 ± 3.55 | 0.464 |
| Trimester 2 Iron (mg) | 253 | 11.27 ± 3.44 | 11.74 ± 3.43 | 11.52 ± 3.44 | 0.281 |
| Trimester 3 Iron (mg) | 254 | 11.48 ± 3.33 | 12.02 ± 3.69 | 11.76 ± 3.52 | 0.225 |
| Trimester 1 Zinc (mg) | 248 | 8.66 ± 2.21 | 8.58 ± 2.42 | 8.62 ± 2.32 | 0.803 |
| Trimester 2 Zinc (mg) | 253 | 8.67 ± 2.41 | 8.54 ± 2.32 | 8.60 ± 2.36 | 0.647 |
| Trimester 3 Zinc (mg) | 254 | 9.08 ± 2.54 | 8.71 ± 2.46 | 8.89 ± 2.50 | 0.234 |
| Trimester 1 Selenium (ug) | 248 | 44.58 ± 16.88 | 43.88 ± 14.81 | 44.21 ± 15.79 | 0.728 |
| Trimester 2 Selenium (ug) | 253 | 45.53 ± 16.51 | 45.98 ± 18.83 | 45.77 ± 17.73 | 0.840 |
| Trimester 3 Selenium (ug) | 254 | 44.60 ± 15.11 | 42.86 ± 15.74 | 43.70 ± 15.43 | 0.368 |
| Trimester 1 Iodine (ug/day) | 248 | 140.13 ± 79.78 | 138.19 ± 55.16 | 139.11 ± 67.76 | 0.823 |
| Trimester 2 Iodine (ug/day) | 253 | 139.62 ± 65.43 | 144.29 ± 64.76 | 142.06 ± 64.99 | 0.569 |
| Trimester 3 Iodine (ug/day) | 254 | 158.24 ± 86.03 | 148.78 ± 60.79 | 153.32 ± 74.00 | 0.310 |
| Trimester 1 Retinol (ug) | 248 | 325.12 ± 139.72 | 335.48 ± 259.38 | 330.59 ± 211.20 | 0.701 |
| Trimester 2 Retinol (ug) | 253 | 314.89 ± 204.59 | 335.20 ± 211.31 | 325.48 ± 207.96 | 0.439 |

**Table S2.** *Cont.*

| **Nutrient** | ***n*** | **Intervention Mean ± SD** | **Control Mean ± SD** | **Total Mean ± SD** | ***p*-Value** |
| --- | --- | --- | --- | --- | --- |
| Trimester 3 Retinol (ug) | 254 | 333.13 ± 150.32 | 344.14 ± 264.57 | 338.85 ± 216.98 | 0.687 |
| Trimester 1 Carotene (ug) | 248 | 3792.55 ± 2428.18 | 3324.54 ± 2473.61 | 3545.33 ± 2458.49 | 0.135 |
| Trimester 2 Carotene (ug) | 253 | 3638.95 ± 2337.05 | 3292.02 ± 3071.62 | 3457.95 ± 2745.11 | 0.316 |
| Trimester 3 Carotene (ug) | 254 | 3789.69 ± 2860.33 | 3652.49 ± 3070.14 | 3718.39 ± 2966.17 | 0.713 |
| Trimester 1 Vitamin D (ug) | 248 | 2.56 ± 1.73 | 2.61 ± 1.65 | 2.59 ± 1.69 | 0.848 |
| Trimester 2 Vitamin D (ug) | 253 | 3.05 ± 2.19 | 2.72 ± 2.30 | 2.88 ± 2.25 | 0.249 |
| Trimester 3 Vitamin D (ug) | 254 | 3.01 ± 2.28 | 2.64 ± 1.79 | 2.82 ± 2.04 | 0.150 |
| Trimester 1 Vitamin E (mg) | 248 | 8.66 ± 3.71 | 7.85 ± 2.91 | 8.23 ± 3.33 | 0.057 |
| Trimester 2 Vitamin E (mg) | 253 | 8.33 ± 3.33 | 8.14 ± 2.77 | 8.23 ± 3.05 | 0.613 |
| Trimester 3 Vitamin E (mg) | 254 | 8.44 ± 3.48 | 8.14 ± 3.52 | 8.28 ± 3.50 | 0.502 |
| Trimester 1 Vitamin K (ug) | 248 | 132.28 ± 86.96 | 124.94 ± 89.16 | 128.40 ± 88.02 | 0.513 |
| Trimester 2 Vitamin K (ug) | 253 | 121.05 ± 84.75 | 116.82 ± 77.57 | 118.84 ± 80.95 | 0.678 |
| Trimester 3 Vitamin K (ug) | 254 | 111.68 ± 73.61 | 127.13 ± 96.46 | 119.71 ± 86.42 | 0.155 |
| Trimester 1 Vitamin C (mg) | 248 | 30.76 ± 32.22 | 37.02 ± 66.17 | 34.07 ± 52.93 | 0.354 |
| Trimester 2 Vitamin C (mg) | 253 | 27.96 ± 30.69 | 30.81 ± 43.93 | 29.45 ± 38.13 | 0.552 |
| Trimester 3 Vitamin C (mg) | 254 | 28.90 ± 38.55 | 25.16 ± 32.31 | 26.96 ± 35.42 | 0.401 |
| Trimester 1 Vitamin B6 (mg) | 248 | 1.59 ± 0.41 | 1.58 ± 0.52 | 1.58 ± 0.47 | 0.895 |
| Trimester 2 Vitamin B6 (mg) | 253 | 1.73 ± 1.85 | 1.62 ± 0.46 | 1.67 ± 1.32 | 0.492 |
| Trimester 3 Vitamin B6 (mg) | 254 | 1.57 ± 0.41 | 1.63 ± 0.47 | 1.60 ± 0.44 | 0.233 |
| Trimester 1 Vitamin B12 (ug) | 248 | 1.70 ± 0.52 | 1.73 ± 0.54 | 1.72 ± 0.53 | 0.661 |
| Trimester 2 Vitamin B12 (ug) | 253 | 1.71 ± 0.58 | 1.76 ± 0.51 | 1.74 ± 0.55 | 0.491 |
| Trimester 3 Vitamin B12 (ug) | 254 | 1.83 ± 0.69 | 1.85 ± 0.54 | 1.84 ± 0.62 | 0.769 |
| Trimester 1 Thiamine (mg) | 248 | 21.26 ± 5.93 | 21.31 ± 7.80 | 21.29 ± 6.97 | 0.962 |
| Trimester 2 Thiamine (mg) | 253 | 21.90 ± 7.09 | 22.26 ± 6.89 | 22.09 ± 6.97 | 0.682 |
| Trimester 3 Thiamine (mg) | 254 | 21.49 ± 6.43 | 22.51 ± 7.34 | 22.02 ± 6.93 | 0.243 |
| Trimester 1 Riboflavin (mg) | 248 | 2.10 ± 0.49 | 2.09 ± 0.76 | 2.10 ± .64 | 0.941 |
| Trimester 2 Riboflavin (mg) | 253 | 2.03 ± 0.55 | 2.18 ± 0.62 | 2.11 ± .59 | 0.045 |

**Table S2.** *Cont.*

| **Nutrient** | ***n*** | **Intervention Mean ± SD** | **Control Mean ± SD** | **Total Mean ± SD** | ***p*-Value** |
| --- | --- | --- | --- | --- | --- |
| Trimester 3 Riboflavin (mg) | 254 | 2.01 ± 0.53 | 2.23 ± 0.63 | 2.12 ± 0.60 | 0.004 |
| Trimester 1 Niacin (mg) | 248 | 4.23 ± 2.04 | 4.49 ± 2.03 | 4.37 ± 2.03 | 0.307 |
| Trimester 2 Niacin (mg) | 253 | 4.45 ± 2.09 | 4.43 ± 2.26 | 4.44 ± 2.18 | 0.946 |
| Trimester 3 Niacin (mg) | 254 | 4.79 ± 2.41 | 4.72 ± 2.04 | 4.75 ± 2.22 | 0.803 |
| Trimester 1 Folate (ug) | 248 | 297.90 ± 96.90 | 277.06 ± 109.86 | 286.89 ± 104.26 | 0.116 |
| Trimester 2 Folate (ug) | 253 | 286.62 ± 97.40 | 272.90 ± 84.16 | 279.46 ± 90.81 | 0.231 |
| Trimester 3 Folate (ug) | 254 | 287.52 ± 105.42 | 289.07 ± 106.00 | 288.32 ± 105.52 | 0.907 |
| Trimester 1 Pantothenic Acid (mg) | 248 | 5.01 ± 1.50 | 4.98 ± 1.41 | 4.99 ± 1.45 | 0.861 |
| Trimester 2 Pantothenic Acid (mg) | 253 | 5.08 ± 1.58 | 5.17 ± 1.44 | 5.13 ± 1.50 | 0.629 |
| Trimester 3 Pantothenic Acid (mg) | 254 | 5.31 ± 1.66 | 5.38 ± 1.36 | 5.34 ± 1.51 | 0.721 |
| Trimester 1 Biotin (ug) | 248 | 28.86 ± 9.78 | 28.04 ± 8.69 | 28.42 ± 9.21 | 0.484 |
| Trimester 2 Biotin (ug) | 253 | 29.69 ± 10.51 | 29.37 ± 10.22 | 29.53 ± 10.34 | 0.809 |
| Trimester 3 Biotin (ug) | 254 | 32.09 ± 13.31 | 30.27 ± 10.61 | 31.15 ± 11.99 | 0.229 |

Independent sample *t*-tests use to compare intervention and control groups. *p*-value < 0.05 considered statistically significant; %TE denotes percentage total energy;
*n* denotes number.

**Table S3.** Maternal characteristics and nutrient intakes associated with neonatal anthropometry unadjusted analysis.

|  | **B** | **SEB** | ***p*** | ***R*^2^** |
| --- | --- | --- | --- | --- |
| **Weight-for-length z-score** |  |  |  |  |
| Trimester 1 sodium | 0.0002 | 0.000095 | 0.027 | 0.016 |
| **Weight-for-age z-score** |  |  |  |  |
| Trimester 2 saturated fat (%TE) | 0.056 | 0.026 | 0.03 | 0.015 |
| **Length-for-age z-score** |  |  |  |  |
| Trimester 1 carotene | 9.14 × 10^−5^ | 0.000034 | 0.008 | 0.025 |
| Mild PA (No. of 20 min intervals/week) | 0.074 | 0.037 | 0.048 | 0.015 |
| Mother height (cm) | 0.012 | 0.006 | 0.05 | 0.011 |
| Trimester 1 carbohydrate (%TE) | −0.031 | 0.013 | 0.019 | 0.019 |
| Trimester 1 total fat (%TE) | 0.031 | 0.015 | 0.042 | 0.013 |
| Trimester 1 polyunsaturated fat (%TE) | 0.133 | 0.044 | 0.003 | 0.033 |
| **BMI ^1^-for-age z-score** |  |  |  |  |
| Trimester 1 sodium | 0.0002 | 0.000095 | 0.03 | 0.016 |
| **Mid-upper-arm circumference-for-age z-score** |  |  |  |  |
| Trimester 1 thiamine | 0.231 | 0.117 | 0.049 | 0.012 |
| Trimester 2 iodine | 0.003 | 0.001 | 0.042 | 0.013 |
| Trimester 2 pantothenate | 0.133 | 0.053 | 0.013 | 0.021 |
| Trimester 2 biotin | 0.016 | 0.008 | 0.045 | 0.012 |
| Trimester 3 sodium | 0.0003 | 0.0001 | 0.011 | 0.023 |
| Trimester 3 iodine | 0.002 | 0.001 | 0.034 | 0.014 |
| Trimester 3 vitamin B6 | 0.443 | 0.185 | 0.018 | 0.019 |
| Trimester 3 vitamin B12 | 0.311 | 0.134 | 0.021 | 0.018 |
| Trimester 3 thiamine | 0.264 | 0.117 | 0.024 | 0.017 |
| Trimester 3 pantothenate | 0.11 | 0.054 | 0.043 | 0.013 |
| Achieved 3rd level education (yes/no) | 0.419 | 0.172 | 0.016 | 0.02 |
| Gestational weight gain (kg) | 0.061 | 0.021 | 0.005 | 0.054 |
| **Triceps skinfold thickness for age z-score** |  |  |  |  |
| Trimester 2 vitamin C | −0.004 | 0.002 | 0.038 | 0.018 |
| Trimester 3 vitamin C | −0.005 | 0.002 | 0.047 | 0.016 |
| Trimester 3 glycaemic Index | 0.063 | 0.021 | 0.003 | 0.045 |
| **Subscapular skinfold thickness for age z-score** |  |  |  |  |
| Trimester 3 riboflavin | −0.302 | 0.147 | 0.041 | 0.017 |
| Moderate PA | 0.084 | 0.042 | 0.048 | 0.03 |
| Met ACOG ^2^ Guidelines | −1.685 | 0.818 | 0.042 | 0.03 |
| **Abdominal circumference** |  |  |  |  |
| Trimester 2 saturated fat (%TE) | 0.184 | 0.079 | 0.021 | 0.017 |
| IOM Guidelines for gestational weight gain (less/met/exceeded) | 0.882 | 0.444 | 0.05 | 0.026 |
| **Thigh circumference** |  |  |  |  |
| Trimester 1 sodium | 0.001 | 0.0003 | 0.023 | 0.017 |
| Trimester 1 carotene | 0.0002 | 0.00008 | 0.027 | 0.016 |
| Trimester 3 sodium | 0.001 | 0.0002 | 0.01 | 0.022 |
| Trimester 3 carbohydrate (%TE) | −0.062 | 0.031 | 0.047 | 0.012 |
| Trimester 3 polyunsaturated fat (%TE) | 0.263 | 0.109 | 0.016 | 0.019 |

**Table S3.** *Cont.*

|  | **B** | **SEB** | ***p*** | ***R*^2^** |
| --- | --- | --- | --- | --- |
| **Chest circumference** |  |  |  |  |
| Baseline strenuous PA (No. of 20 min intervals/week) | 1.359 | 0.493 | 0.011 | 0.209 |
| Gestational weight gain (kg) | 0.107 | 0.049 | 0.03 | 0.027 |
| Glucose booking (mmol/L) | 0.946 | 0.474 | 0.047 | 0.011 |
| Child gender | −1.762 | 0.328 | 0 | 0.091 |
| **Hip circumference** |  |  |  |  |
| Gestational weight gain (kg) | 0.153 | 0.068 | 0.027 | 0.028 |
| **Biceps skinfold** |  |  |  |  |
| Trimester 1 selenium | 0.024 | 0.009 | 0.01 | 0.029 |
| Trimester 1 vitamin B6 | 0.826 | 0.32 | 0.011 | 0.028 |
| Trimester 1 thiamine | 0.651 | 0.194 | 0.001 | 50 |
| Trimester 1 riboflavin | 0.448 | 0.223 | 0.046 | 0.015 |
| Baseline No. minutes sitting/weekday | 0.002 | 0.001 | 0.042 | 0.017 |
| Trimester 3 glycaemic index | 0.124 | 0.034 | 0 | 0.062 |
| Trimester 1 protein (%TE) | 0.093 | 0.047 | 0.046 | 0.015 |
| Trimester 1 total fat (%TE) | −0.054 | 0.025 | 0.037 | 0.017 |
| Trimester 1 monounsaturated fat (%TE) | −0.136 | 0.058 | 0.019 | 0.023 |
| **Thigh skinfold** |  |  |  |  |
| Trimester 1 iodine | 0.009 | 0.003 | 0.003 | 0.04 |
| Trimester 1 vitamin B6 | 0.995 | 0.461 | 0.032 | 0.019 |
| Trimester 1 riboflavin | 0.773 | 0.318 | 0.016 | 0.025 |
| Trimester 2 magnesium | 0.007 | 0.003 | 0.019 | 0.023 |
| Trimester 2 thiamine | 0.711 | 0.284 | 0.013 | 0.026 |
| Trimester 2 riboflavin | 0.686 | 0.331 | 0.04 | 0.016 |
| Mother MUAC booking (cm) | 0.158 | 0.063 | 0.014 | 0.025 |
| Gestational weight gain (kg) | −0.106 | 0.045 | 0.019 | 0.041 |
| Trimester 3 polyunsaturated fat (%TE) | 0.3 | 0.116 | 0.011 | 0.028 |
| **Sum of all skinfolds** |  |  |  |  |
| Trimester 3 glycaemic index | 0.266 | 0.102 | 0.01 | 0.029 |
| **Sum of subscapular and triceps skinfolds** |  |  |  |  |
| Trimester 2 carotene | −0.0002 | 0.00007 | 0.026 | 0.02 |
| Trimester 2 vitamin C | −0.014 | 0.005 | 0.009 | 0.03 |
| Trimester 3 riboflavin | −0.938 | 0.353 | 0.008 | 0.03 |
| Attend Gym (yes/no) | −1.297 | 0.645 | 0.046 | 0.015 |
| Trimester 3 glycaemic index | 0.122 | 0.051 | 0.017 | 0.024 |
| **Subscapular:triceps skinfold ratio** |  |  |  |  |
| Trimester 1 sodium | −5.36 × 10^−5^ | 0.00002 | 0.015 | 0.025 |
| Trimester 3 glycaemic index | −0.011 | 0.004 | 0.01 | 0.029 |
| Gestational weight gain (kg) | 0.014 | 0.005 | 0.003 | 0.072 |
| Gestation (days) | 0.005 | 0.002 | 0.046 | 0.016 |
| Met ACOG^2^ Guidelines (yes/no) | −0.43 | 0.171 | 0.013 | 0.046 |

**Table S3.** *Cont.*

|  | **B** | **SEB** | ***p*** | ***R*^2^** |
| --- | --- | --- | --- | --- |
| **Waist:length circumference ratio** |  |  |  |  |
| Trimester 3 calcium | 2.01 × 10^−5^ | 0.00001 | 0.037 | 0.013 |
| Trimester 3 iodine | 0.0001 | 0.00004 | 0.01 | 0.022 |
| Trimester 3 retinol | 3.00 × 10^−5^ | 0.00001 | 0.038 | 0.013 |
| Duration of breastfeeding (months) | −0.003 | 0.002 | 0.036 | 0.033 |
| Mild PA (No. of 20 min intervals/week) | 0.003 | 0.001 | 0.015 | 0.023 |
| Mother hours/day watching television | −0.011 | 0.005 | 0.043 | 0.013 |
| Trimester 3 saturated fat (%TE) | 0.002 | 0.001 | 0.039 | 0.013 |
| Trimester 1 polyunsaturated fat (%TE) | −0.005 | 0.002 | 0.002 | 0.034 |
| IOM Guidelines for gestational weight gain (less/met/exceeded) | 0.011 | 0.005 | 0.05 | 0.021 |
| **Waist:hip circumference ratio** |  |  |  |  |
| Trimester 1 vitamin B6 | −0.022 | 0.01 | 0.032 | 0.015 |
| Trimester 3 potassium | 1.48 × 10^−5^ | 0.000007 | 0.029 | 0.015 |
| Trimester 3 iodine | 0.0001 | 0.00006 | 0.039 | 0.013 |
| Trimester 1 glycaemic index | −0.002 | 0.001 | 0.031 | 0.015 |
| Glucose booking (mmol/L) | 0.027 | 0.013 | 0.036 | 0.013 |
| Trimester 1 carbohydrate (%TE) | −0.002 | 0.001 | 0.011 | 0.022 |
| Trimester 1 total fat (%TE) | 0.002 | 0.001 | 0.007 | 0.026 |
| Trimester 1 saturated fat (%TE) | 0.005 | 0.002 | 0.001 | 0.038 |
| Trimester 2 saturated fat (%TE) | 0.004 | 0.002 | 0.01 | 0.022 |
| Trimester 2 polyunsaturated fat (%TE) | −0.006 | 0.003 | 0.024 | 0.016 |

Simple Linear Regression was used for this analysis (significant associations included). *p*-value < 0.05 was considered statistically significant; ^1^ BMI denotes body mass index; ^2^ ACOG denotes American Congress of Obstetricians and Gynecologists. Abbreviations: No.: number; %TE: percentage of total energy; IOM: Institute of Medicine; MUAC: Mid-upper arm circumference; PA: physical activity; SEB: the standard error of the computed value of B.
